# Vaginal Hysterotomy

**Published:** 1880-08

**Authors:** 


VAGINAL HYSTEROTOMY.—Our former townsman, Prof. E. W. Jenks, now of the Chicago Medical College, has successfully performed the difficult and rare operation of vaginal hysterotomy, removing the entire uterus through the vagina. The operation was performed on the 8th ult., and the patient has been discharged completely recovered. The operation was resorted to on account of malignant disease of the uterus, and was the only procedure which could shed a ray of hope on a hopeless disease. We congratulate our friend Jenks on his success, which, however, can only serve to establish more securely, if possible, the position which merit has long since secured for him among the gynecologists of this country.—*Therapeutic Gazette.*

